# Complete genome sequence of *Spirochaeta smaragdinae* type strain (SEBR 4228^T^)

**DOI:** 10.4056/sigs.1143106

**Published:** 2010-10-18

**Authors:** Konstantinos Mavromatis, Montri Yasawong, Olga Chertkov, Alla Lapidus, Susan Lucas, Matt Nolan, Tijana Glavina Del Rio, Hope Tice, Jan-Fang Cheng, Sam Pitluck, Konstantinos Liolios, Natalia Ivanova, Roxanne Tapia, Cliff Han, David Bruce, Lynne Goodwin, Amrita Pati, Ami Chen, Krishna Palaniappan, Miriam Land, Loren Hauser, Yun-Juan Chang, Cynthia D. Jeffries, John C. Detter, Manfred Rohde, Evelyne Brambilla, Stefan Spring, Markus Göker, Johannes Sikorski, Tanja Woyke, James Bristow, Jonathan A. Eisen, Victor Markowitz, Philip Hugenholtz, Hans-Peter Klenk, Nikos C. Kyrpides

**Affiliations:** 1 DOE Joint Genome Institute, Walnut Creek, California, USA; 2 HZI – Helmholtz Centre for Infection Research, Braunschweig, Germany; 3 Los Alamos National Laboratory, Bioscience Division, Los Alamos, New Mexico, USA; 4 Biological Data Management and Technology Center, Lawrence Berkeley National Laboratory, Berkeley, California, USA; 5 Oak Ridge National Laboratory, Oak Ridge, Tennessee, USA; 6 DSMZ - German Collection of Microorganisms and Cell Cultures GmbH, Braunschweig, Germany; 7 University of California Davis Genome Center, Davis, California, USA

**Keywords:** spiral shaped, corkscrew-like motility, chemoorganotroph, strictly anaerobe, obligately halophile, rhodanese-like protein, *Spirochaetaceae*, GEBA

## Abstract

*Spirochaeta smaragdinae* Magot *et al*. 1998 belongs to the family *Spirochaetaceae*. The species is Gram-negative, motile, obligately halophilic and strictly anaerobic and is of interest because it is able to ferment numerous polysaccharides. *S. smaragdinae* is the only species of the family *Spirochaetaceae* known to reduce thiosulfate or element sulfur to sulfide. This is the first complete genome sequence in the family *Spirochaetaceae*. The 4,653,970 bp long genome with its 4,363 protein-coding and 57 RNA genes is a part of the *** G****enomic* *** E****ncyclopedia of* *** B****acteria and* *** A****rchaea * project.

## Introduction

Strain SEBR 4228^T^ (= DSM 11293 = JCM 15392) is the type strain of the species *Spirochaeta smaragdinae*. Currently, there are eighteen species [1] and two subspecies in the genus *Spirochaeta* [1,2]. The generic name derives from the Greek word ‘*speira*’ meaning ‘a coil’ and the Greek word ‘*chaitê*’ meaning ‘hair’, referring to the spiral shape of bacterial cell. The species epithet is derived from the Latin word ‘*smaragdinae*’ meaning ‘from Emerald’, referring to the name Emerald of an oil field in Congo. Strain SEBR 4228^T^ was isolated from an oil-injection production water sample of a Congo offshore oilfield [3] and described in 1997 by Magot *et al*. as ‘*Spirochaeta smaragdinae*’ [3]. Here we present a summary classification and a set of features for *S. smaragdinae* SEBR 4228^T^, together with the description of the complete genomic sequencing and annotation.

## Classification and features

Strain SEBR 4228^T^ shares 82.2-99.0% 16S rRNA gene sequence identity with the type strains from the other members of genus *Spirochaeta* [4], with the type strain of *S. bajacaliforniensis* [5], isolated from a mud sample in Laguna Figueroa (Baja California, Mexico) showing the highest degree of sequence similarity (99%). Notwithstanding the high degree of 16S rRNA gene sequence identity, these two strains are characterized by low genomic similarity (38%) in DNA-DNA hybridization studies and differ by numerous differences in carbon source utilization [3]. Several type strains from the genus *Treponema* show the highest degree of similarity for non-*Spirochaeta* strains (82.9-83.6%) [4]. A representative genomic 16S rRNA sequence of strain SEBR 4228^T^ was compared using BLAST with the most recent release of the Greengenes database [6] and the relative frequencies of taxa and keywords, weighted by BLAST scores, were determined. The three most frequent genera were *Spirochaeta* (76.4%), ‘*Sphaerochaeta*’ (15.8%) and *Cytophaga* (7.8%). Within the five most frequent keywords in the labels of environmental samples were 'microbial' (11.7%), 'mat' (10.5%), 'hypersaline' (7.7%), and 'sediment' (1.7%). The environmental samples database (env_nt) contains the marine metagenome genomic clone 1061006082084 (EK988302) that is 92% identical to the 16S rRNA gene sequence of SEBR 4228^T^. No phylotypes from genomic surveys could be linked to the species *S. smaragdinae* or even the genus *Spirochaeta*, indicating a rather rare occurrence of these in the habitats screened so far (as of August 2010).

**Figure 1:**
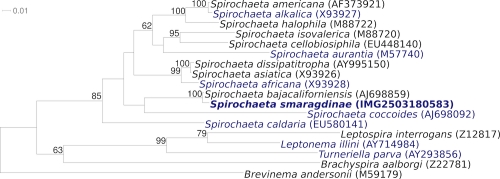
Phylogenetic tree highlighting the position of *S. smaragdinae* SEBR 4228^T^ relative to the type strains of the other species within the genus and of the other genera within the genus *Spirochaeta*. The tree was inferred from 1,385 aligned characters [7,8] of the 16S rRNA gene sequence under the maximum likelihood criterion [9] and rooted in accordance with the current taxonomy [10]. The branches are scaled in terms of the expected number of substitutions per site. Numbers above branches are support values from 500 bootstrap replicates [11] if larger than 60%. Lineages with type strain genome sequencing projects registered in GOLD [12] are shown in blue, published genomes in bold.

Figure 1 shows the phylogenetic neighborhood of *S. smaragdinae* SEBR 4228^T^ in a 16S rRNA based tree. The sequences of the two 16S rRNA gene copies differ from each other by up to one nucleotide, and differ by up to five nucleotides from the previously published 16S rRNA sequence generated from DSM 11293 (U80597), which contains two ambiguous base calls.

**Figure 2:**
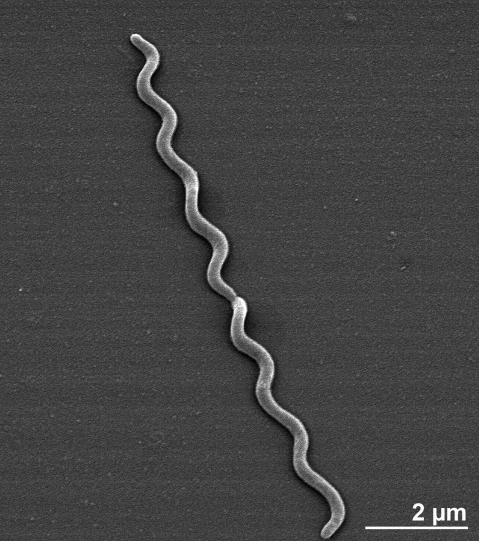
Scanning electron micrograph of *S. smaragdinae* SEBR 4228^T^

Strain SEBR 4228^T^ is a Gram-negative, chemoorganotrophic and strictly anaerobic bacterium with spiral shaped, 0.3-0.5 × 5-30 μm long cells (Figure 2 and Table 1). It possesses a multilayer, crenulating, Gram-negative cell envelope, which consists of an outer membrane and an inner membrane adjoining the cytoplasmic membrane [3]. Sillons, which are the contact point between the protoplasmic cylinder, the inner membrane and the outer membrane, are also observed from the cells of *S. smaragdinae* SEBR 4228^T^ [3]. Strain SEBR 4228^T^ forms translucent colonies with regular edges (0.5 mm of diameter) after two weeks of incubation on SEM agar plates at 37°C [3]. The strain is motile with a corkscrew-like motion, which is characteristic for the typical 1-2-1 periplasmic flagellar arrangement of the members of the genus *Spirochaeta* [3]. The periplasmic, non-extracellular location of the flagella make the *Spirochaeta* a valuable candidate for the study of flagella evolution [26]. The enlarged spherical bodies, which are typical for spirochetes, are also observed in strain SEBR 4228^T^ [3]. The temperature range for growth is from 20°C to 40°C, with an optimum temperature at 37°C [3]. The pH range for growth is between 5.5 and 8.0, with an optimum pH of 7.0 [3]. Strain SEBR 4228^T^ is obligately halophilic [3] and is able to grow on media that contains 1-10% of NaCl, with an optimum salinity at 5% NaCl [3]. Under optimum growth conditions, the doubling time is approximately 25 h in the presence of glucose and thiosulfate [3]. Strain SEBR 4228^T^ is able to utilize biotrypcase, fructose, fumarate, galactose, D-glucose, glycerol, mannitol, mannose, ribose, D-xylose and yeast extract, but not acetate, D-arabinose, butyrate, casamino acids, lactate, maltose, propionate, pyruvate, rhamnose, sorbose, sucrose and L-xylose [3]. Yeast extract is required for growth and cannot be replaced by a vitamin mixture [3]. Strain SEBR 4228^T^ ferments fumarate to acetate and succinate [3]. The major end-product of glucose fermentation of strain SEBR 4228^T^ is lactate with traces of H_2_ and ethanol [3]. *S. smaragdinae* is the only species of *Spirochaeta* known to reduce thiosulfate or elemental sulfur to sulfide [3]. Strain SEBR 4228^T^ produces lactate, acetate, CO_2_ and H_2_S as the end-products of glucose oxidation when thiosulfate is present in the growth medium [3]. The strain contains a rhodanese-like protein which expresses rhodanese activity [27]. This enzyme is able to reduce thiosulfate to sulfide [28]. Rhodanese is also widely found in other members of the domain *Bacteria* [29-31].

**Table 1:** Classification and general features of *S. smaragdinae* SEBR 4228^T^ according to the MIGS recommendations [13].

**MIGS ID**	**Property**	**Term**	**Evidence code**
	Current classification	Domain *Bacteria*	TAS [14]
		Phylum *Spirochaetae*	TAS [15,16]
		Class *Spirochaetes*	TAS [16]
		Order *Spirochaetales*	TAS [17,18]
		Family *Spirochaetaceae*	TAS [18,19]
		Genus *Spirochaeta*	TAS [18,20-22]
		Species *Spirochaeta smaragdinae*	TAS [3,23]
		Type strain SEBR 4228	TAS [3]
	Gram stain	negative	TAS [3]
	Cell shape	spiral	TAS [3]
	Motility	yes	TAS [3]
	Sporulation	none	NAS
	Temperature range	between 20°C and over 40°C	TAS [3]
	Optimum temperature	37°C	TAS [3]
	Salinity	1-10% NaCl (optimum 5%)	TAS [3]
MIGS-22	Oxygen requirement	obligately anaerobic	TAS [3]
	Carbon source	polysaccharides	TAS [3]
	Energy source	chemoorganotroph	TAS [3]
MIGS-6	Habitat	oil-fields	TAS [3]
MIGS-15	Biotic relationship	free-living	TAS [3]
MIGS-14	Pathogenicity	none	NAS
	Biosafety level	1	TAS [24]
	Isolation	oil-injection water sample in the production system of an oil field	TAS [3]
MIGS-4	Geographic location	Emerald oil fields in Congo	TAS [3]
MIGS-5	Sample collection time	1997 or before	TAS [3]
MIGS-4.1	Latitude	not reported	
MIGS-4.2	Longitude	not reported	
MIGS-4.3	Depth	not reported	
MIGS-4.4	Altitude	not reported	

Evidence codes - IDA: Inferred from Direct Assay (first time in publication); TAS: Traceable Author Statement (i.e., a direct report exists in the literature); NAS: Non-traceable Author Statement (i.e., not directly observed for the living, isolated sample, but based on a generally accepted property for the species, or anecdotal evidence). These evidence codes are from of the Gene Ontology project [25]. If the evidence code is IDA, then the property was directly observed by one of the authors or an expert mentioned in the acknowledgements

### Chemotaxonomy

No cellular fatty acids profiles are currently available for *S. smaragdinae* SEBR 4228^T^. However, C_16:0_ dimethyl acetate is the major cellular fatty acids of the type strains of the closely related *S. dissipatitropha*, *S. asiatica* and *S. americana*, and C_16:0_ fatty acid methyl ester is the major cellular fatty acids of *S. africana* [20,32].

## Genome sequencing and annotation

### Genome project history

This organism was selected for sequencing on the basis of its phylogenetic position [33], and is part of the *** G****enomic* *** E****ncyclopedia of* *** B****acteria and* *** A****rchaea * project [34]. The genome project is deposited in the Genome OnLine Database [12] and the complete genome sequence is deposited in GenBank. Sequencing, finishing and annotation were performed by the DOE Joint Genome Institute (JGI). A summary of the project information is shown in Table 2.

**Table 2:** Genome sequencing project information

**MIGS ID**	**Property**	**Term**
MIGS-31	Finishing quality	Finished
MIGS-28	Libraries used	Three genomic libraries: 454 pyrosequence standard and PE (12 kb insert size) libraries and one Illumina standard library
MIGS-29	Sequencing platforms	454 GS FLX Titanium, Illumina GAii
MIGS-31.2	Sequencing coverage	58.8 × pyrosequence, 6.9 × Illumina
MIGS-30	Assemblers	Newbler version 2.0.0-PostRelease- 11/04/2008, phrap,
MIGS-32	Gene calling method	Prodigal 1.4, GenePRIMP
	INSDC ID	CP002116
	Genbank Date of Release	August 6, 2010
	GOLD ID	Gc013354
	NCBI project ID	32637
	Database: IMG-GEBA	2503128010
MIGS-13	Source material identifier	DSM 11293
	Project relevance	Tree of Life, GEBA

### Growth conditions and DNA isolation

*S. smaragdinae* SEBR 4228^T^, DSM 11293,  was grown anaerobically in medium 819 (*Spirochaeta smaragdinae* medium) [35] at 35°C. DNA was isolated from 0.5-1 g of cell paste using MasterPure Gram Positive DNA Purification Kit (Epicentre MGP04100) following the standard protocol as recommended by the manufacturer, with modification st/LALMice for cell lysis as described in Wu *et al.* [34].

### Genome sequencing and assembly

The genome was sequenced using a combination of Illumina and 454 sequencing platforms. All general aspects of library construction and sequencing can be found at the JGI website (http://www.jgi.doe.gov/). Pyrosequencing reads were assembled using the Newbler assembler version 2.0.0-PostRelease-11/04/2008 (Roche). The initial Newbler assembly consisted of 51 contigs in one scaffold was converted into a phrap assembly by making fake reads from the consensus, collecting the read pairs in the 454 paired end library. Illumina GAii sequencing data was assembled with Velvet [36] and the consensus sequences were shredded into 1.5 kb overlapped fake reads and assembled together with the 454 data. Draft assemblies were based on 273 Mb 454 draft data and all of the 454 paired end data. Newbler parameters are -consed -a 50 -l 350 -g -m -ml 20.

The Phred/Phrap/Consed software package (www.phrap.com) was used for sequence assembly and quality assessment in the following finishing process. After the shotgun stage, reads were assembled with parallel phrap (High Performance Software, LLC). Possible mis-assemblies were corrected with gapResolution (http://www.jgi.doe.gov/), Dupfinisher, or sequencing cloned bridging PCR fragments with subcloning or transposon bombing (Epicentre Biotechnologies, Madison, WI) [37]. Gaps between contigs were closed by editing in Consed, by PCR and by Bubble PCR primer walks (J.-F.Chang, unpublished). A total of 147 additional reactions were necessary to close gaps and to raise the quality of the finished sequence. Illumina reads were also used to improve the final consensus quality using an in-house developed tool - the Polisher [38]. The error rate of the completed genome sequence is 0.2 in 100,000. Together, the combination of the Illumina and 454 sequencing platforms provided 65.7× coverage of the genome.

### Genome annotation

Genes were identified using Prodigal [39] as part of the Oak Ridge National Laboratory genome annotation pipeline, followed by a round of manual curation using the JGI GenePRIMP pipeline [40]. The predicted CDSs were translated and used to search the National Center for Biotechnology Information (NCBI) nonredundant database, UniProt, TIGRFam, Pfam, PRIAM, KEGG, COG, and InterPro databases. Additional gene prediction analysis and functional annotation was performed within the Integrated Microbial Genomes - Expert Review (IMG-ER) platform [41].

## Genome properties

**Figure 3:**
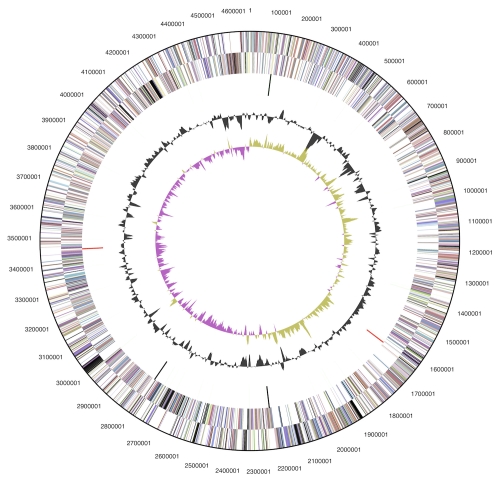
Graphical circular map of the genome. From outside to the center: Genes on forward strand (color by COG categories), Genes on reverse strand (color by COG categories), RNA genes (tRNAs green, rRNAs red, other RNAs black), GC content, GC skew.

The genome consists of a 4,653,970 bp long chromosome with a 49.0% GC content (Table 3 and Figure 3). Of the 4,363 genes predicted, 4,306 were protein-coding genes, and 57 RNAs; eighty seven pseudogenes were also identified. The majority of the protein-coding genes (74.2%) were assigned with a putative function while the remaining ones were annotated as hypothetical proteins. The distribution of genes into COGs functional categories is presented in Table 4.

**Table 3:** Genome Statistics

**Attribute**	**Value**	**% of Total**
Genome size (bp)	4,653,970	100.00%
DNA coding region (bp)	4,315,215	92.97%
DNA G+C content (bp)	2,278,823	48.97%
Number of replicons	1	
Extrachromosomal elements	0	
Total genes	4,363	100.00%
RNA genes	57	1.31%
rRNA operons	2	
Protein-coding genes	4306	98.69%
Pseudo genes	87	1.99%
Genes with function prediction	3,235	74.15%
Genes in paralog clusters	818	18.75%
Genes assigned to COGs	3,318	76.05%
Genes assigned Pfam domains	3,443	78.91%
Genes with signal peptides	871	26.36%
Genes with transmembrane helices	1,150	22.45%
CRISPR repeats	1	

**Table 4:** Number of genes associated with the general COG functional categories

**Code**	**Value**	**%age**	**Description**
J	159	4.3	Translation, ribosomal structure and biogenesis
A	0	0.0	RNA processing and modification
K	328	8.8	Transcription
L	129	3.5	Replication, recombination and repair
B	1	0.0	Chromatin structure and dynamics
D	25	0.7	Cell cycle control, cell division, chromosome partitioning
Y	0	0.0	Nuclear structure
V	58	1.6	Defense mechanisms
T	321	8.6	Signal transduction mechanisms
M	183	4.9	Cell wall/membrane/envelope biogenesis
N	94	2.5	Cell motility
Z	0	0.0	Cytoskeleton
W	0	0.0	Extracellular structures
U	58	1.6	Intracellular trafficking and secretion, and vesicular transport
O	114	3.1	Posttranslational modification, protein turnover, chaperones
C	223	6.0	Energy production and conversion
G	553	14.9	Carbohydrate transport and metabolism
E	326	8.8	Amino acid transport and metabolism
F	96	2.6	Nucleotide transport and metabolism
H	130	3.5	Coenzyme transport and metabolism
I	61	1.6	Lipid transport and metabolism
P	165	4.4	Inorganic ion transport and metabolism
Q	30	0.8	Secondary metabolites biosynthesis, transport and catabolism
R	450	12.1	General function prediction only
S	212	5.7	Function unknown
-	1,045	23.9	Not in COGs
